# Injectable Biologics for the Treatment of Degenerative Disc Disease

**DOI:** 10.1007/s12178-020-09668-2

**Published:** 2020-07-23

**Authors:** Ajay Matta, W. Mark Erwin

**Affiliations:** 1 Notogen Inc, 661 University Ave., Suite 1300, Toronto, Ontario M5G 0B7 Canada; 2 Divisions of Orthopedic and Neurological Surgery, University of Toronto, 661 University Ave., Suite 1300, Toronto, Ontario M5G 0B7 Canada

**Keywords:** Intervertebral disc disease, Back pain, Stem cell treatment, Disc injections

## Abstract

**Purpose of Review:**

Spinal pain and associated disability is a leading cause of morbidity worldwide that has a strong association with degenerative disc disease (DDD). Biologically based therapies to treat DDD face significant challenges posed by the unique milieu of the environment within the intervertebral disc, and many promising therapies are in the early stages of development. Patient selection, reasonable therapeutic goals, approach, and timing will need to be discerned to successfully translate potential therapeutics. This review provides a brief overview of the status of intradiscal biologic therapies.

**Recent Findings:**

Proposed systemic delivery of therapeutic agents has not progressed very much in large part due to the risk of adverse events in remote tissues plus the very limited vascular supply and therefore questionable delivery to the intervertebral disc nucleus pulposus. Intradiscal delivery of therapeutic proteins shows good potential for clinical trials and translation with encouraging results from large animal pre-clinical studies plus an enhanced understanding of the biology of DDD. There are a few cell-based therapies currently under pre-clinical and clinical trial investigation; however, these attempts continue to be hampered by unknown if any, mechanism of action, no downstream detection of transplanted cells, mixed results concerning efficacy, small sample numbers, and a lack of objective evidence of pain mediation.

**Summary:**

Treatment of DDD using biologically based therapeutics is a widely sought-after goal; however, potential therapies need to address pain and disability in larger, well-controlled studies.

## Introduction

Back and neck pain secondary to degenerative disc disease (DDD) is the health condition with the highest economic cost to society, with direct and indirect costs estimated to range between $19.6 and $118.8 billion in the USA [1–3]. Epidemiologic studies have reported that spinal pain is the leading cause of years lost to disability worldwide with degenerative disc disease (DDD) accounting for a large proportion of back pain [3, 4]. DDD results in the loss of cellularity, structural integrity, biomechanical properties, and height of the intervertebral disc that can contribute to pain, instability, and deformity [5]. Since there is no cure and there are controversial roles for surgery restricted to cases of instability or deformity, current treatments for DDD are limited to symptom management, such as physical therapies, anti-inflammatory medications, and analgesics. The intervertebral disc (IVD) nucleus pulposus (NP) variably contains stem cells (nucleus pulposus progenitor cells or “NPPCs”), chondrocyte-like cells (CLCs), notochordal cells (NCs) (in youth in humans and in some animals such as rats, rabbits, and non-chondrodystrophic dogs), and fibroblasts, collectively termed “NP” cells. DDD is a multifactorial condition, but trauma, aging, genetics, and occupational stresses are factors that lead to a catabolic cascade of increased inflammatory cytokines and extracellular matrix (ECM) degrading molecules/enzymes resulting in excessive inflammation and progressive tissue damage [5, 6]. The degenerative intervertebral disc (IVD) loses cellularity, structural integrity, and biomechanical properties and changes in nociception, biological changes that can variably contribute to pain, instability, and deformity [5]. Impaired biological regulation of the IVD as described above can lead to motor unit (the two vertebrae and disc lying in between) overload and secondary pain arising from inflammation/dysfunction of the segmental facet joints, joint capsules, and associated musculature [7]. Therefore, new interventions including biologics and/or tissue engineering approaches are currently under intense investigation with a view to being able to influence the course of the disorder [8].

## Therapeutic Interventions

The intervertebral disc is largely avascular apart from the peripheral annulus, whereas the inner annulus and nucleus pulposus are hypoxic, ischemic, aneural, and isolated from the immune system. The IVD is sandwiched between the upper and lower vertebral end plates that, in addition to loading platforms, act to control the diffusion of nutrients, waste products, and gases principally from the IVD NP into and out of the vertebral body. It has been reported that progressive DDD involves calcification of the tiny pores within the endplate resulting in impaired diffusion and compromise of the gas/nutrient exchange that in turn furthers the degenerative cascade [9, 10]. Therefore, to be effective, any biological intervention must be able to mitigate the harsh, pro-inflammatory, pro-catabolic, and anti-anabolic environment within the degenerative disc.

## Systemic Therapies

Oral or injectable therapeutics that may address DDD face considerable challenges in that they must first undergo absorption without degradation and then transport into the largely avascular, ischemic IVD. Interestingly, a number of manuscripts have been published detailing the use of systemic treatments for DDD such as the use of melatonin, Celastrol, or glucosamine sulfate [11–13]. In the melatonin study, the investigators reported that there may be a role played by melatonin in the development/progression of DDD in that pinealectomy has been shown to accelerate DDD in some vertebrates and that melatonin acts via specific cell surface receptors [11]. The conclusions of the study were that melatonin is a crucial regulator of NP cell function and that in vitro treatment of human IVD NP cells with melatonin downregulated extracellular matrix remodeling enzymes, increased collagen type 2 and aggrecan expression, and decreased cellular proliferation. The study demonstrated in vitro evidence that melatonin can influence NP cells and may provide a hypothetical model for how pinealectomy may influence DDD; this study was limited to an in vitro approach, and the translational potential for such therapy remains obscure. Another systemic approach to treat DDD has been published concerning the use of “Celastrol,” a traditional Chinese medicine used in the treatment of a host of illnesses from diabetes to obesity, atherosclerosis, hearing loss, cancer, and neurodegenerative conditions [12]. The possible mechanism(s) of action appear to be principally via suppressing the pro-inflammatory pathway by inhibiting activation of NF-κB as well as mediating the activity of JNK kinases and caspase mediators of apoptosis [12]. Limiting the use of Celastrol is the low bioavailability and relatively narrow toxicity range of the drug as well as any reasonable route of administration. In the study by Chen et al., the drug was administered via intraperitoneal injection daily for up to 6-week post-needle puncture injury prior. Although the investigators report favorable anti-degenerative effects of treatment as well as favorable results using human IVD NP cells in vitro, the translational aspects of this kind of therapy are challenging. Systemic administration would require that the concentration of the agent must meet therapeutic levels within the hypoxic, ischemic, and avascular IVD without meeting systemic toxic levels. Amino sugars such as glucosamine sulfate/hydrochloride have been postulated to confer benefit to degenerative joint disease such as osteoarthritis, with conflicting results. An in vivo study examining the use of oral glucosamine supplementation in a rabbit model of DDD showed that injured animals treated with oral glucosamine demonstrated an anti-anabolic effect [13]. The ability of any systemically applied intervention to treat DDD must overcome the challenges of the relatively avascular, ischemic, and isolated IVD NP with little progress over the past few decades, leading to rise of the intradiscal approach of direct injection of the therapeutic agent.

## Biomolecular Injection

The direct injection of biomolecules into the IVD NP including anabolic/anti-catabolic proteins has been extensively studied particularly over the past decade. Growth factors encompass a broad range of pro-anabolic biomolecules that generally increase cellular proliferation, cellular viability, and beneficial effects upon the extracellular matrix. There are a multitude of growth factors known, some of which lie within “super families” such as the transforming growth factor superfamily (TGF). Within the overall umbrella of the TGF superfamily are the bone morphogenetic proteins (BMPs) (including BMP-2, BMP-7, BMP-12), as well as other BMP sub-families like GDF-5/-6, as well as TGF-β1 and TGF-β3 among others. There are other biomolecules molecules including insulin-like growth factor (IGF), platelet-derived growth factor (PDGF), epidermal growth factor (EGF), as well as a poorly defined “cocktail” of factors contained within platelet-rich plasma (PRP)[14]and/or catabolic enzyme inhibitors, all of which have been attempted as therapeutic interventions to treat DDD [8].

**Fig. 1:**
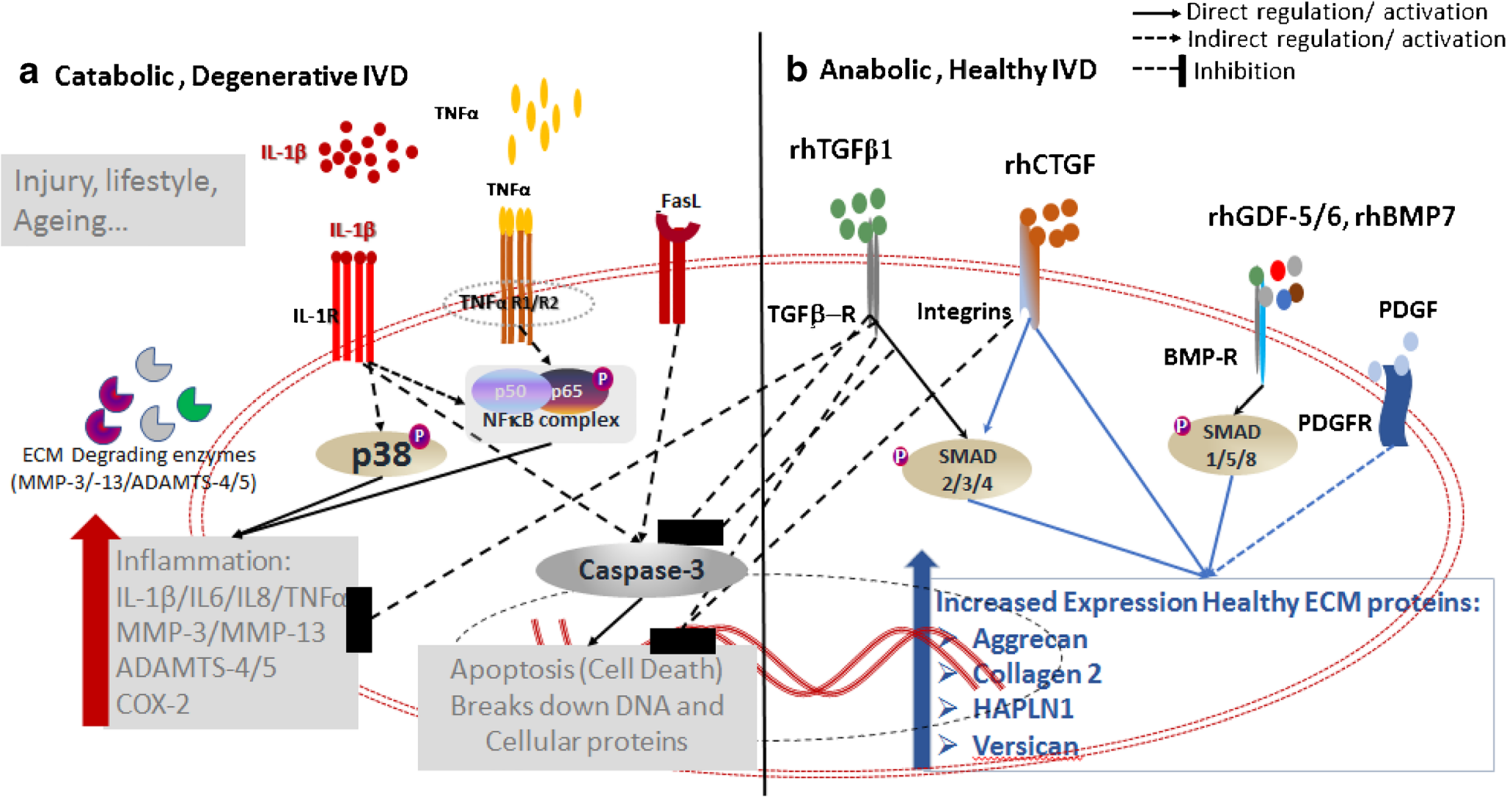
Role of biologics therapy for degenerative disc disease (DDD). Schematic representing (**a**) pro-catabolic, degenerative micro-environment in IVD with increased expression of pro-inflammatory cytokines (IL-1β, TNFα) and death ligand (FasL) because of injury, aging, or lifestyle changes. These pro-inflammatory cytokines activate nuclear factor kappa B (NF-κB) and p38MAPK signaling pathways inducing matrix metalloproteinases (MMP-3/MMP-13), aggrecanases (ADAMTS-4/5), pain-associated protein cyclooxygenase 2 (Cox2) in degenerative IVDs; (**b**) An intradiscal biologic therapy includes growth factors (rhTGFβ-1/3, rhCTGF, rhGDF-5/6, rhBMP-7, PDGF), PRP, or cellular replacement therapy. Treatment with a combination of rhTGFb1 and rhCTGF inhibits inflammation-induced signaling, thereby mitigating inflammation and inducing pro-anabolic, healthy ECM proteins (aggrecan, Collagens) both in vitro and in vivo animal models of DDD. Treatment with other growth factors including rhGDF-5/6, rhBMP-7, and PDGF has been reported to induce expression of healthy extracellular matrix proteins in IVD

With respect to the delivery of growth factors, a study involving the injection of BMP-7, also known as OP-1, into a rabbit model of DDD was reported 16 years ago, and this procedure reportedly led to an increase in disc height with an improved elastic modulus [15]. Another study in which BMP-2 was injected into degenerative rabbit discs led to worsened degeneration with enhanced vascularization and fibroblast proliferation [16]. However, a follow-up study using a canine model of DDD and an intradiscal administration of recombinant human (rh)BMP-7 not only did not show a therapeutic benefit, but it caused undesired bone formation external to the IVD [17]. These two reports underscore important aspects with respect to the injection of a putative therapeutic protein. The salient aspect concerning the injection of putative therapeutic agents is an understanding of the mechanisms of action of the growth factor and their respective signaling. For example, GDF-5/6, sub-classes of BMPs, themselves members of the TGF superfamily, signal through specific surface receptors that activate highly conserved intracellular signaling proteins, in this case, known as “Smads.” BMPs signal via Smad-1, 5, and 8 whereas TGF-β1 and TGF-β3 signal via Smad-2, 3, and 4. These respective signaling cascades are of vital importance with respect to their downstream activation of dependent genes. The BMPs, by definition, are bone-inducing molecules that although also having proliferation potential and some beneficial aspects regarding extracellular matrix synthesis and also induce the formation of bone. This is the clear conclusion in the study involving injection of BMP-7 (OP-1) in the canine study. The Smad-2, 3, and 4 intracellular signaling pathways do not lead to bone formation but rather upregulate cell survival, ECM synthesis, and downregulation of pro-inflammatory signaling. Specifically, TGF-β1 downregulates pro-catabolic signaling induced by pro-inflammatory cytokines as IL-1β, TNFα, IL-6, and IL-8, all of which are major players in progressive degenerative disc disease (Fig. 1).

These signaling events are tightly regulated and involve interplay with other mediators which in the case of BMPs include both canonical and non-canonical signaling that involve MAP kinases, Wnts, Akt/mTOR microRNAs, and importantly Runx2 as possibly a key integrative signaling molecule [18]. The non-canonical pathways involve in Smad-independent signaling also implicate P38 MAP kinases that in turn can activate Runx2 that is central to osteoblastic differentiation and bone formation. It is vital to understand these signaling pathways to avoid undesirable bone formation as was seen following injection of BMP-7/OP-1 in the canine study cited above. TGF-β1 on the other hand signals via the Smad-2/3/4 pathway that does not implicate Runx2 and osteogenesis [18]. In the studies published by Matta et al., there were impressive anti-degenerative effects and no reports of untoward bone formation [19, 20].

Published accounts have suggested that growth factor delivery would need to be repeated and have high doses of the therapeutic protein in order to provide any benefit [8, 21]. Theoretically, activity of endogenous proteases within the IVD may degrade injected therapeutic proteins, and/or the persistence and bioavailability of injected biomolecules may be transient. However, recent published data refute this suggestion in that Matta et al. demonstrated in both rat and chondrodystrophic canines that a single injection of recombinant human connective tissue growth factor (rhCTGF) and recombinant human transforming growth factor beta-1 (rhTGF-β1) suspended within an excipient solution showed ongoing transcription activity at least 14 weeks post-injection in canines as well as preservation of disc height and retained biomechanical properties compared with vehicle (saline) injections [20]. It is therefore unknown whether therapeutic protein delivery has only a transient activity particularly if the proteins are delivered within a formulation that enhances the bioavailability of the proteins. Some have advocated the use of slow release formulation or vector transmission in order to increase the duration of molecular delivery to the target tissue [8]. With respect to increasing the therapeutic effect of biomolecule delivery, Yan et al. investigated GDF-5 packaged in microspheres that reportedly enabled the slow release of GDF-5 for over 42 days. This approach reportedly demonstrated some improvement in a rat-tail model of DDD in terms of increased disc height, sulfated glycosaminoglycan content, and improved histological scores [22]. With respect to longer term bioactivity, Tellegen et al. recently published an account where celecoxib-loaded microspheres were injected in a pre-clinical canine model and reported that the release of celecoxib was observed for over 28 days; however, there was no meaningful regenerative effect [23]. Nonetheless, the notion of enhancing delivery of a biomolecular therapy using sustained, slow-release mechanisms may have utility if dose, concentration, and pharmacokinetics can be appropriately controlled and validated.

### Therapeutic Gene Delivery

There have been numerous accounts of attempts to deliver genes that encode therapeutic proteins into the IVD to potentiate the presence of these proteins and mediate potential limitations of direct therapeutic protein delivery. Several possible vectors including adenovirus, adeno-associated virus (AAV), retrovirus, baculovirus, and lentiviruses have been investigated. Although some of these gene delivery systems have shown promising results in pre-clinical investigations, safety is an overriding concern [24••]. Limitations to these approaches include the lack of integration of the adenoviral vector into the host DNA, which although reducing mutation risk in the protein expressed by the virus is expressed transiently. AAV only carries a limited amount of genetic information and carries with it difficulties in introduction into the target cells [25]. Nonetheless, therapeutic gene delivery faces challenges with safety due to a study using rabbits in which a higher dose of adeno-associated viral vectors expressing TGFβ1 and rhBMP-7 induced bilateral lower limb paralysis clearly indicating the potential risk of this approach [26]. Furthermore, undesirable transgene-induced protein production may lead to unintended adverse events indicating that the gene therapy approach as traditionally applied may provide more risk than benefit [27••]. With respect to gene-based approaches, there have been recent attempts at the use of CRISPR-Cas9 gene editing to knock out and/or repair dysfunctional gene regulation. However, these approaches are very early and largely limited to in vitro experimentation [28••].

### Platelet-Rich Plasma

Platelet-rich plasma (PRP) is a method whereby a small volume of concentrated activated platelets is injected into an area of injury and logically extends the idea of growth factor-based therapy since the mechanism of action of PRP is almost certainly due to the growth factor content of the injectate that can activate tissue repair [29, 30]. In vitro experimentation has shown that PRP can stimulate cell proliferation and induce proteoglycan and glycosaminoglycan synthesis in soft tissue such as tendon and muscle [31]. A pre-clinical study using PRP in rat-tail models of DDD provided some evidence of anti-degenerative effects in the animals that received a PRP injection immediately, post injury, or 2 weeks later and showed some reduced degeneration; however, animals that received the injections 6 weeks post injury developed much more degenerative changes [32]. These results are interesting in that the rat disc is highly notochordal, and notochordal cells have been shown to confer anti-degenerative effects on IVD NP cells; therefore, it would be difficult to determine the beneficial effects of an acute injection of any therapeutic as compared with notochordal cell-based activity when the injection occurs within the acute or early sub-acute period. Matta et al. determined that the resolution of needle puncture injury matures by 10 weeks post injury resulting in significant loss of notochordal and stem cells in intervertebral disc nucleus pulposus [19, 20]. Overall, there have been several studies evaluating in vitro and in vivo injection of PRP as a potential DDD therapy; however, there is considerable inconsistency using in vivo pre-clinical animal models [33]. A recent review by Akeda et al. has shown that although there have been in vitro and some in vivo (with mixed results) studies concerning PRP injections to treat DDD, only one double-blind randomized controlled trial has been completed that showed significant improvements in functional rating scale, patient satisfaction, and numerical rating scale (NRS) worst pain at 8-week follow-up. The lack of no treatment control group beyond 8 weeks severely limits conclusions of this study. The authors reported that most of the treated patients were followed for 1 year with “beneficial effects” with respect to the FRI Index (measures participation perception of function and pain related to performing dynamic movements and holding static positions). Nonetheless, Oswestry disability or VAS scores were not reported [29, 34]. Surprisingly, no imaging studies were reported for the study patients. Also, only 56% of the patients reported being satisfied with the treatment. Potential confounders/limitations to the use of PRP are the lack of standardization of dose, large donor variability, method of preparation, and the lack of understanding with respect to purported mechanism of action. Although the study results are interesting, it is difficult to imagine a hypothetical mechanism for the reported improvements in only 8 weeks, and if “most of the treated patients” reported beneficial effects 1 year later also in the absence of imaging, one is left to wonder about the results with no control group beyond 8 weeks. Furthermore, with respect to the notion of intradiscal biotherapeutics, there has been explanation or even hypothesis concerning how the effects of a single PRP injection may confer long-term benefits or how pain relief as reported by Tuakli-Wosornu et al. may be conferred within 8 weeks.

### Cellular-Replacement Therapy

In addition to the catabolic, pro-inflammatory changes that occur within the degenerative disc, DDD is also known to involve a loss of viable cells [35–37]. Therefore, cellular replacement strategies have emerged as potential methods to mitigate against the progressively acellular disc as a possible regenerative therapy. Several potential cell types have been postulated as candidates for cellular replacement including fibroblasts, bone marrow, adipose, umbilical cord-derived stem cells, IVD NP cells, or disc-derived chondrocytes. Some of these cellular replacement therapeutics have been and continue to be studied in clinical trials following limited pre-clinical studies. However, much remains to be determined concerning the use of cellular replacement.

#### Mechanism of Action

There is scant evidence for the mechanism of action of transplanted cells given the wide variety of cells used for this purpose. For example, dermal-derived fibroblasts have been investigated as a potential source; however, in this approach, the desire is to alter the cellular configuration of the IVD into one of a connective tissue rather than confer a regenerative strategy. A recent publication concerning the use of dermal fibroblast as possible cellular transplants purported to show that such transplants conferred restorative effects upon Cynomolgus monkeys. In this study, six young monkeys that received surgical exposure and following needle puncture were either injected with dermal fibroblasts or sham controls (details lacking with respect to the sham procedure). The authors reported that the animals injected with the fibroblasts demonstrated retained IVD height as compared with needle puncture and hypothesize that the treatment resulted in a reparative fibrosis repair [38]. Of note, the fibroblast transplants occurred immediately at the time of disc injury with no time interval between needle puncture injury and cell therapy. Further, the treatment groups were only followed for 8 weeks, a very short period of time from which to draw meaningful conclusions. Another limitation to the study pertained to the phenotypic and TGF-β and Smad signaling that the authors reported to be induced by fibroblasts. The authors used rat IVD NP cells co-cultured with fibroblasts and deduced that fibroblastic cells can induce NP cells to adopt a more fibroblastic phenotype [38]. Monolayer culture is known to induce fibroblastic differentiation, and rat-tail IVDs contain an almost 100% notochordal cellular phenotype, and normoxic (21% O_2_) tissue culture is inconsistent with the intradiscal environment. Finally, the MRI data concerning the Cynomolgus monkey study had very broad variability and few numbers of animals leading to difficulty in arriving at convincing data.

Although not a cellular therapy, others have published with respect to injecting the degenerative disc with a formulation including lactic acid to induce a fibrous, connective tissue “healing.” A single ascending dose study of the “STA363” compound has been completed in 15 patients with chronic discogenic low back pain (August 2019); however, no results have yet been posted (https://clinicaltrials.gov/ct2/show/results/NCT03055845).

### Stem Cells

One of the most studied sources of stem cells is bone marrow (stromal) cell-sourced stem cells (BMSCs) that have been the subject of numerous clinical trials. Historically, clinical trials involving BMSCs have been of small sample size and lack adequate controls and a continued lack of description of mechanism of action or evidence that the transplanted cells actually integrate into the NP [39]. A study published by Henriksson et al. concerning the traceability of mesenchymal stromal cells injected into the discs of only 4 patients resulted in all patients subsequently undergoing spinal fusion surgery [40••]. However, post-surgery, the IVD tissue removed was investigated for the presence of iron sucrose that was pre-labeled in the MSCs. The investigators were able to detect iron sucrose 8 months post-transplantation, and it was claimed that the cells had differentiated into chondrocyte-like cells [40••]. It is significant in that this study is among the only trials that demonstrate the presence of cells post-transplant over the longer term; however, all subjects progressed to require spinal fusion surgery post-transplant, yielding the questionable utility of such transplants in the mitigation of DDD, let alone any regenerative capacity. Other clinical trials involving the injection of up to 25 × 10^6^ BMSCs or between 6 and 18 × 10^6^ cells yield further evidence that there is no consensus with respect to the number of cells that ought to be transplanted (https://clinicaltrials.gov/ct2/show/record/NCT01860417), (https://clinicaltrials.gov/ct2/show/NCT01290367).

## Conclusions

Over the past decade, considerable progress has been in our understanding of the mechanisms involved with the development and progression of DDD. These advances have led to the evolution of novel candidate therapies and, in some cases, clinical trials; however, none have yet achieved the desired goal that convincingly modulating the course of DDD. Among the obstacles posed to biological therapy for the degenerative disc are the reduced pH, avascular status, chronic inflammation, and progressive cell death that occur with DDD. Overcoming these challenges will require a minimally invasive intervention that can activate repair by endogenous IVD NP cells with or without cellular replacement that is active over the longer term. Most importantly, the therapy will need to positively impact pain of discogenic origin, a challenge that has a constellation of driving forces. It is therefore necessary to understand the contributions of biological factors that drive discogenic pain, which along with other patient-specific determinants summate in the full clinical picture. To this end, it is also important to keep expectations of what intradiscal therapy might realistically achieve at the forefront in the development of these yet to come innovative therapeutics.

## Abbreviations

AAV: Adeno-associated virus
BMP-7: Bone morphogenetic protein-7
BMP-R: Bone morphogenetic protein receptor
CTGF: Connective tissue growth factor
DDD: Degenerative disc disease
ECM: Extracellular matrix
EGF: Epidermal growth factor
GDF-5/6: Growth and differentiation Factor-5/6
IGF: Insulin growth factor
IVD: Intervertebral Disc
NC: Notochordal cells
NF-κB: Nuclear Factor kappa B
NP: Nucleus pulposus
NPPCs: Nucleus pulposus progenitor cells
OP-1: Osteogenic protein-1
PDGF: Platelet-derived growth factor
PDGFR: Platelet-derived growth factor receptor
PRP: Platelet-rich plasma
rh: Recombinant human
CLCs: Chondrocyte-like cells
IL-1β: Interleukin-1 beta
IL-6: Interleukin-6
IL-8: Interleukin-8
TGF-1/3: Transforming growth factor beta-1/3
TGFβR: Transforming growth factor beta receptor
TNFα: Tumor necrosis factor alpha
